# Novel Rearrangements in the Staphylococcal Cassette Chromosome *Mec* Type V Elements of Indian ST772 and ST672 Methicillin Resistant *Staphylococcus aureus* Strains

**DOI:** 10.1371/journal.pone.0094293

**Published:** 2014-04-10

**Authors:** Jayanth Balakuntla, Sushma Prabhakara, Gayathri Arakere

**Affiliations:** Society for Innovation and Development, Indian Institute of Science, Bangalore, India; Rockefeller University, United States of America

## Abstract

*Staphylococcus aureus* is a commensal gram positive bacteria which causes severe and non severe infections in humans and livestock. In India, ST772 is a dominant and ST672 is an emerging clone of *Staphylococcus aureus*. Both cause serious human diseases, and carry type V SCC*mec* elements. The objective of this study was to characterize SCC*mec* type V elements of ST772 and ST672 because the usual PCR methods did not amplify all primers specific to the type. Whole genome sequencing analysis of seven ST772 and one ST672 *S. aureus* isolates revealed that the SCC*mec* elements of six of the ST772 isolates were the smallest of the extant type V elements and in addition have several other novel features. Only one ST772 isolate and the ST672 isolate carried bigger SCC*mec* cassettes which were composites carrying multiple *ccrC* genes. These cassettes had some similarities to type V SCC*mec* element from M013 isolate (ST59) from Taiwan in certain aspects. SCC*mec* elements of all Indian isolates had an inversion of the *mec* complex, similar to the bovine SCC*mec* type X. This study reveals that six out of seven ST772 *S. aureus* isolates have a novel type V (5C2) SCC*mec* element while one each of ST772 and ST672 isolates have a composite SCC*mec* type V element (5C2&5) formed by the integration of type V SCC*mec* into a MSSA carrying a SCC element, in addition to the *mec* gene complex inversions and extensive recombinations.

## Introduction

*Staphylococcus aureus* is an important pathogen in hospitals and in communities causing a broad range of infections among both humans and animals. Treatment of severe infections is becoming a challenge due to development of multi-drug resistance. Methicillin resistance appeared soon after the introduction of this antibiotic in 1960. It is due to the presence of *mecA* gene coding for the protein PBP2A, which has a low affinity for β-lactam antibiotics [1]–[4]. The *mecA* gene is housed within a unique mobile genetic element known as Staphylococcal Cassette Chromosome *mec* (SCC*mec*) integrated in the staphylococcal genome. The SCC*mec* is comprised of (1) *mec* gene complex containing the *mecA* gene, its regulatory genes and associated insertion sequences, and (2) cassette chromosome recombinase (*ccr*) gene complex [5], [6]. Six classes of *mec* gene complexes (A, B, C1, C2, D and E) and three *ccr* genes (*ccrA, ccrB* and *ccrC*) for integration and excision of the SCC*mec* element have been reported so far (www.sccmec.org). SCC*mec* elements are classified into types by the combination of the type of *ccr* gene complex and the class of *mec* gene complex. Eleven SCC*mec* elements are reported to date: SCC*mec* I to XI [5]–[8]. Among these, SCC*mec* types I–V are the most commonly reported. SCC*mec* types I–III are usually carried by hospital- associated methicillin resistant *S. aureus* (HA-MRSA) while types IV and V are usually carried by community-associated (CA) MRSA. However, now the distinction between HA and CA MRSA is getting blurred [9].

The first isolate to be characterized with SCC*mec* element V(5C2) containing *ccrC* was *S.aureus* strain WBG8318 (WIS) belonging to ST45 from Australia. The next was TSGH17 from Taiwan with ST59 genetic background and with two *ccrCs* (5C2&5) [10]–[12]. Since then, many MRSA isolates such as M013 with SCC*mec* V elements containing two *ccrCs*, have been identified in various genetic backgrounds and with different *ccrC1* alleles [13], [14].

ST772, known as the Bengal Bay clone, prevalent in Bangladesh, India, and Malaysia, is a single locus variant of ST1 in *pta* [15]–[18]. International travel has spread this clone to Japan and several European countries [19], [20]. A recent German study has shown complex skin and soft tissue infections to be associated with Panton-Valentine Leukocidin (PVL) positive ST772 *S. aureus* among individuals returning from Asia [21]. The PVL-positive ST772 carrying SCC*mec* element V is one of the predominant clones present in Indian hospitals and the community, and is known to cause serious diseases [16], [17]. A PVL-negative ST672 clone carrying SCC*mec* element V is also circulating among carriers and patients in India [22]. ST672 *S. aureus*, from Western Australia, carrying SCC*mec* elements IVa and V, is designated as a single locus variant of ST361 by Coombs et al. [23]. Very few ST672 isolates have been reported in the MLST database.

Two factors lead us to believe that the type V SCC*mec* elements of ST772 and ST672 could be different from each other and that of strain WIS. Firstly, primers identifying the *ccrC* and C2-*mec* gene complexes amplified while none of the joining (J) region primers amplified and secondly, during microarray analysis of SCC*mec* type V Indian isolates, ST772 and ST672 MRSA isolates tested positive for probe containing the *ccrAA*(MRSAZH47) region, while isolates belonging to ST8 were negative. This lead us to perform whole genome sequencing of 7 isolates of ST772 from different clinical backgrounds and one isolate of ST672 [16], [17], [22], [24]. By identifying the SCC*mec* regions from whole genome sequences, we have shown that ST772 and ST672 SCC*mec* elements have novel rearrangements compared to extant type V elements.

## Results and Discussion

We chose seven ST772 isolates and one ST672 *S. aureus* isolate from different clinical backgrounds for whole genome sequencing. Table 1 shows details of Indian isolates investigated in the present study and other reference strains used in comparison of genomes of SCC*mec* elements along with their accession numbers.

**Table 1:** Clinical history, molecular characterization and accession numbers of sequenced and reference isolates.

Isolate/Strain	Place/Source	Clinical History	Year of isolation	ST/CC	PVL	*Agr* type	Gen Bank Accession No^a^	DDBJ Accession No^b^	SCC*mec* size (bp)	No of ORFs
118	Bangalore / blood	Pyomyositis	2008	772/1	+	II	AJGE00000000	AB777516	25,389	26
VH60	Bangalore/nasal swab	Carrier	2007	772/1	+	II	ALWG00000000	AB781450	25,396	28
3989	Hyderabad/sputa	Pneumonitis	2007	772/1	+	II	ALWH00000000	AB781447	24,870	27
120	Bangalore/pus	Cerebral Abscess	2009	772/1	+	II	ALWE00000000	AB781444	25,288	26
333	Madurai/corneal ulcer	Endophthalmitis	2010	772/1	+	II	ALWF00000000	AB781445	26,528	26
LVP2	Bhubaneshwar/keratitis	Microbial keratitis	2010	772/1	+	II	AOFV00000000	AB781449	24,512	26
3957	Hyderabad/pus	Breast abscess	2007	772/1	+	II	AOFU00000000	AB781446	36,199	39
GR1	Delhi/blood	Septicemia	2007	672/361	-	I	AJLX00000000	AB781448	34,776	40
M013^c^	Taiwan/	Wound Infection	2002	59	+	I	CP003166	-	41,265	39
WIS^d^	Australia	Skin carriage	1995	45/45	-		-	AB121219	28,612	25
JCSC6945^e^	Denmark	Carrier	2006	398				AB505653.1	51,483	54
85/2082^f^	Newzealand		1985	239/8	-	I		AB037670.1	68,256	80

a : Whole genome sequenced contigs;

b : Annotations for the SCC*mec* region;

c Huang TW, Chen F, Miu WC, Liao TL, Lin AC, et al. (2012) J. Bacteriol 194:1256-1257;

d : O'Brien FG, Coombs GW, Pearson JC, Christiansen KJ, Grubb WB. (2005) Antimicrob. Agents Chemother 49: 5129-5132;

e: :Li S, Skov RL, Han X, Larsen AR, Larsen J, et al. (2011) Antimicrob. Agents Chemother 55: 3046-3050;

f : Ito, T., Y. Katayama, K. Asada, N. Mori, K. Tsutsumimoto, C. Tiensasitorn, and K. Hiramatsu. 2001. Antimicrob. Agents Chemother. 45: 1323-1336.

We identified the SCC*mec* elements from whole genome sequences by using previously published "Chromosome"-SCC*mec* junction sequences at the SCC integration site. Six of the seven ST772 isolates with the exception of *S. aureus* 3957, carried SCC*mec* elements ranging in length from 24512 bp (LVP2) to 26528 bp (333). These are the smallest type V SCC*mec* elements so far reported with the number of open reading frames (orfs) varying between twenty six and twenty eight.

*S. aureus* 3957 contains the largest ST772 SCC*mec* element with 39 orfs. Table 2 presents data comparing the orfs present in the SCC*mec* element of 3957 to corresponding orthologs of one of the six similar ST772 isolates (118), ST672 isolate (GR1) and two reference strains WIS and M013 (representative of 5C2 and 5C2&5 SCC*mec* elements respectively). A similar Table S1 reports on the orfs present in the SCC*mec* element of *S. aureus* VH60 (highest number of orfs) in comparison with all the other ST772, ST672 and reference strains. While there is 100% identity in the sequence of rRNA methyl transferase (orfX), the hypothetical proteins (HPs) coded by orfs 2 – 8 and orfs 10 – 13 present in 3957 are not identified in 118. Two HPs coded by orfs 7 and 15 in VH60 are not identified in most of the isolates (Table S1). The sequenced isolates came from different clinical backgrounds and it is apparent that there are differences in HPs, insertional sequences and transposases.

**Table 2:** Comparison of orfs from SCC*mec* elements of S. *aureus* 3957 and corresponding orthologs of other ST772, ST672 and reference strains.

3957 (ST772)			Homology (%) a									
			118 (ST772)		GR1 (ST672)		WIS (ST45)		M013 (ST59)		Others	
Orfs	Position	Predicted Function	% Identity	C O^b^	% Identity	C O	% Identity	C O	% Identity	C O	% Identity	C O
orf 1	369..848, 160 aa	rRNA methyltransferase	100	118.1	100	GR1.1	100	BAD24821.1	100	YP_005296500.1		
orf 2	1094..1399, 102 aa	HP^c^	NI^f^		100	GR1.2	NI		100	YP_005296501.1		
orf 3	1598..246, 288 aa	HP	NI		100	GR1.3	NI		100	YP_005296502.1		
orf 4	2569..4044, 492 aa	HP	NI		100	GR1.4	NI		100	YP_005296504.1		
orf 5	4270..5370, 367 aa	HP (repair, recombination and replication)	NI		100	GR1.5	77.13	BAD24831.1	100	YP_005296505.1		
orf 6	5363..5734, 124 aa	HP	NI		100	GR1.6	81.15	BAD24832.1	100	YP_005296506.1		
orf 7	5731..7374, 548 aa	Putative Primase	NI		100	GR1.7	81.38	BAD24833.1	100	YP_005296507.1		
orf 8	7367..7444, 26 aa	HP	NI		100	GR1.8	NI		100	YP_005296508.1		
orf 9	7600..9276, 559 aa	CcrC1 (allele 8)	95.2	118.17	99.82	GR1.9	94.61	BAD24834.1	100	YP_005296509.1		
orf 10	9382..9720, 113 aa	HP	NI		100	GR1.10	49.55	BAD24835.1	NI		100 (ZH47)	CAL22884.1
orf 11	9722..9823, 34 aa	HP	NI		100	GR1.11	NI		NI		97 (*S. epidermis*)	EJE32225.1
orf 12	9816..10127, 104 aa	HP	NI		100	GR1.12	50.94	BAD24837.1	100	YP_005296510.1		
orf 13	10143..10649, 169 aa	DUF1643 superfamily protein	NI		100	GR1.13	65.87	BAD24838.1	100	YP_005296511.1		
orf 14	11277..10609,223 aa	IS431 mec	95.6	118.3	98.9	GR1.14	99.04	BAD24823.1	100	YP_005296512.1		
orf 15	11589..13595, 669 aa	Penicillin binding protein PBP2'	100	118.4	100	GR1.15	100	BAD24826.1	100	YP_005296516.1		
orf 16	14069..13641, 143 aa	HP	100	118.5	100	GR1.16	100	BAD24825.1	100	YP_005296515.1		
orf 17	14909..14166, 248 aa	ugpQ^d^	100	118.6	100	GR1.17	100	BAD24824.1	100	YP_005296514.1		
orf 18	15993..15826, 56 aa	HMG-CoA	100	118.7	100	GR1.19	NI		100	YP_005296513.1		
orf 19	16251..16334, 28 aa	HP	NI		NI		NI		NI		88 (USA300-TCH959)	ZP_04865955.1
orf 20	16377..17696, 440 aa	Transposase for IS1181	NI		NI		NI		NI		99 (*S. aureus* A9781)	ZP_05642705.1
orf 21	18368..17940, 143 aa	PhnB like proteins^e^	100	118.9	99.3	GR1.21	99.3	BAD24829.1	99.3	YP_005296517.1		
orf 22	18449..19378, 310 aa	HP (transcriptional regulation response to unknown ligand)	100	118.10	100	GR1.22	NI		100	YP_005296518.1		
orf 23	19540..21528, 663 aa	HP	100	118.11	100	GR1.23	99.85	BAD24830.1	99.8	YP_005296522.1		
orf 24	21723..22832, 370 aa	HP (Family A polymerase functions in DNA repair)	100	118.12	100	GR1.24	96.75	BAD24831.1	100	YP_005296523.1		
orf 25	22825..23193, 123 aa	HP	100	118.14	100	GR1.26	65.57	BAD24832.1	100	YP_005296524.1		
orf 26	23193..24809, 539 aa	HP (distant relative to ccr)	100	118.15	100	GR1.27	77.09	BAD24833.1	100	YP_005296525.1		
orf 27	24802..24879, 26 aa	HP (IS-125)	100	118.16	100	GR1.28	NI		100	YP_005296508.1		
orf 28	25034..25285, 84 aa	Truncated ccrC (serine recombinase domain)	NI		97.59	GR1.29	89.16	BAD24834.1	96.37	YP_005296526.1		
orf 29	25282..26718, 479 aa	ccrC	97.07	118.17	100	GR1.29	94.97	BAD24834.1	97.49	YP_005296526.1		
orf 30	26807..27145, 113 aa	HP	98.21	118.18	100	GR1.31	91.96	BAD24835.1	99.11	YP_005296527.1		
orf 31	27148..27237, 30 aa	HP	NI		100	GR1.32	NI		NI		100 (HP)	EJD84594.1
orf 32	27239..27550, 104 aa	HP	100	118.19	100	GR1.33	87.38	BAD24837.1	85.44	YP_005296528.1		
orf 33	27568..28071, 168 aa	HP	100	118.20	100	GR1.34	92.81	BAD24838.1	95.21	YP_005296529.1		
orf 34	28085..28306, 74 aa	HP	100	118.21	100	GR1.35	NI		93.15	YP_005296530.1		
orf 35	31499..28380,1040aa	hsdR (type I restriction enzyme R protein)	100	118.22	100	GR1.36	96.54	BAD24840.1	97.02	YP_005296531.1		
orf 36	32640..31483, 386 aa	hsdS (type I restriction modification DNA specificity domain)	100	118.23	100	GR1.37	36.56	BAD24841.1	34.72	YP_005296532.1	80 (*S. aureus* LGA251)	YP_005754070.1
orf 37	34144..32630, 505 aa	hsdM (type I restriction modification DNA methyltransferase subunit M)	100	118.24	100	GR1.38	96.23	BAD24842.1	98.21	YP_005296533.1		
orf 38	34329..35204, 292 aa	HP (nucleotidyltransferase domain of 2'5'-oligoadenylate synthetase)	100	118.25	100	GR1.39	NI		NI		98 (*S.epidermidis* W23144)	ZP_04797658.1
orf 39	35428..35976, 183 aa	HP	100	118.26	100	GR1.40	NI		NI		99 (*S.epidermidis* W23144)	ZP_04797657.1

a Identity of the amino acid sequence to each ortholog (orf);

b Corresponding Ortholog in the reference strain,

C Hypothetical Protein;

d ugpQ: glycerophosphoryl diester phosphodiesterase;

e PhnB-like proteins adopting structural fold similar to bleomycin resistance proteins;

f No Identity.

### Small-sized SCC*mec* elements carried by six ST772 strains

**Figure 1:**
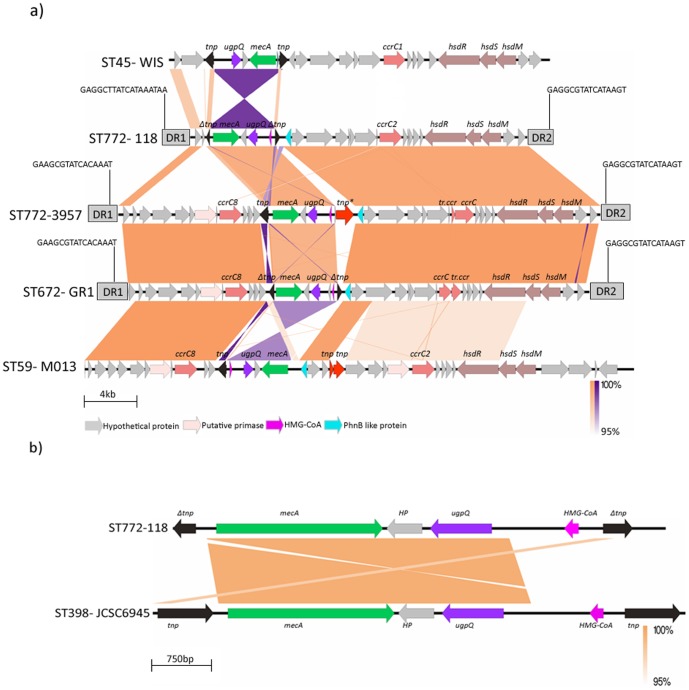
Schematic representation of genetic structures of type V SCC*mec* elements. a) Schematic representation and comparison of genetic structures of type V (5C2) SCC*mec* element of 118 (ST772), and type V (5C2&5) SCC*mec* elements of 3957 (ST772) and GR1(ST672) and the reference strains WIS (ST45) and M013 (ST59). Structures of these elements are illustrated based on nucleotide sequences deposited in DDBJ/EMBL/GenBank database under the accession numbers AB777516, AB781446, AB781448, AB121219 and CP003166. Coding sequences are marked in the direction of transcription as arrows. Transposases for IS431 are indicated in black arrows. Intact transposases for IS431 have been labeled as *tnp*, truncated transposases for IS431 have been labeled as *Δtnp*. Tranposase for IS1181and transposase for M013 are indicated in red arrows and have been labeled as tnp* and *tnp*, respectively. Color coding for the genes not labeled are shown in the legend. Conserved region with more than 95% homology are indicated in light brown and *mec* gene complex inversions are shown in purple as determined by BLASTN. Genomic picture was generated using Easy Fig software. 1b: Comparison of *mec* gene complexes in S. *aureus* 118 and JCSC6945.

*OrfX* insertion site and the characteristic terminal inverted and direct repeats, generated upon insertion of SCC*mec*, were almost similar to extant SCC*mec* elements. Genomic maps and comparison of SCC*mec* elements of strains WIS, 118 (representing six smaller ST772 SCC*mec* elements), 3957, GR1 and M013 are illustrated in Figure 1a drawn using Easy Fig software [25]. The differences and similarities between the genomic structures of various SCC*mec* elements are highlighted below.

Sequence analysis of this region from Figure 1a and Table 2 reveals large differences in HPs, IS431 transposases and the C2*mec* gene complex between WIS and 118, between 118 and 3957, and between 3957 and GR1 and M013. All sequenced isolates contain the class C2 *mec* gene complex; the arrangement of genes downstream of *orfX* between the two *IS431mec* transposases however, are significantly different. In the SCC*mec* elements of ST772 and ST672 isolates, there are inversions in the *mec* gene complex with the absence of *mecR1/ Δ mecR*. This inversion is similar to the inversion reported in isolate JCSC6945 (ST398) collected from a Canadian participant in an international Pig Veterinary Conference. JCSC6945 contains SCC*mec* element X with C1 *mec* gene complex, while ST772 and 672 isolates carry SCC*mec* element V with a C2 *mec* gene complex [7]. Figure 1b depicts similar *mec* gene complex inversions in strain 118 and JCSC6945 but with a different orientation of IS431 which might have occurred during a horizontal transfer. *S. aureus* ST398 is associated with livestock and human infections and is an important pathogen [26], [27]. SCC*mec* type V element has been characterized recently in UMCG-M4, a ST398 human isolate containing PVL but does not harbour *mec* gene complex inversion [28].

Downstream of *mecA*, next to the second IS431, our isolates and M013, unlike WIS, contain a PhnB-like protein, which adopts structural folds similar to bleomycin resistance protein. The HPs downstream of the PhnB-like protein are similar among ST772, and ST672 isolates.

All the six isolates have similar arrangements with minor variations in HPs, IS431 transposases and non coding regions. IS431 transposases of all six isolates located at both upstream and downstream of the *mecA* gene are truncated to different extents as shown in Table S2. IS431 transposases (represented as *Δtnp* in figure 1) located at the downstream of *mecA* in strains VH60, 118, 333, 3989 and LVP2 are truncated to the same size (48 aa), while 120 has a larger sized *Δtnp* (157 aa). Similarly, IS431 transposases (represented as *Δtnp* in figure 1) located at the upstream of *mecA* in two isolates from eye infections, 333 and LVP2, have larger *Δtnps* (133, 155 aa respectively) while the *Δtnps* of 60, 118, 120 and 3989 are of same size (92 aa) and smaller. *S. aureus* 118 and all the other 5 isolates have a *ccrC1*(allele 2) downstream of the *mec* gene complex while WIS has *ccrC1*(allele 1). All six ST772 isolates and WIS carry SCC*mec* elements type V (5C2) although with many differences. It is evident that WIS and these 772 isolates have evolved independently. To our knowledge, no other type V (5C2 or 5C2&5) SCC*mec* element has been reported with inversion of the *mec* complex.

### SCC*mec* elements of S. aureus 3957, and GR1

While GR1 has two *Δtnps* of equal size, 3957 has an IS431 transposase at the upstream of *mecA* and an IS similar to IS1181 having a transposase of 440 amino acids long. Similar partial IS431 transposases have been found in *S. aureus* TSGH17 and ZH47. *S. aureus* 3957 and GR1contain the same *mec* gene complex inversion as in other ST772 isolates and, in addition, type 5 *ccr* gene complex comprising of *ccrC1* (allele 8) in the region between *orfX* and *mec* gene complex. *S. aureus* 3957 and GR1 carry two other truncated (split) *ccrCs*, downstream of *mec* gene complex, split into two due to a frame shift mutation. Orfs 28 and 29 of 3957 and orfs 29 and 30 of GR1 have 98, 97, 100 and 95% identity respectively with ccrC1 (allele 5) from *S. haemolyticus* JCSC1435 (YP_251971.1) which is an intact protein of 558 amino acids carrying serine recombinase, zinc finger, and flxA domains. In GR1 split ccrCs are encoded by orf 29 (311 amino acids) and 30 (247 amino acids) carrying two recombinase domains (one serine recombinase and zinc finger domain, respectively) while in 3957, they are encoded on orf 28 (83 amino acids as initial part of serine recombinase) and orf 29 (478 amino acids) with latter part of serine recombinase, resolvase, zinc finger and flxA domains respectively. ClustalW alignments of the split ccrCs of *S. aureus* 3957 and GR1 with *ccrC1*(allele 5) sequences are shown in Figure S1. The designations for the truncated *ccrCs* have to await the decision from the International Working Group on SCC*mec* elements.

The type V SCC*mec* elements of *S. aureus* 3957 and GR1 are composite cassettes (5C2&5) formed by the integration of type V SCC*mec* element into a SCC element of a MSSA isolate and have in all probability evolved independently from the more common 5C2 ST772 isolates.

SCC*mec* element in *S. aureus* 3957 is an exception to other ST772 isolates as it carries the region similar to M013 in GR1 (ST672). To check the frequency of appearance of SCC*mec* elements similar to 3957, we screened 45 ST 772 isolates from our collection with primers specific for 3957 and did not find any other ST772 isolate with a unique SCC*mec* element found in 3957. Hence it appears that the recombination event is not frequent although it is stable in this isolate. ST672 may not be highly prevalent for the same reason.

### Restriction-Modification systems

Like other type V SCC*mec* elements, ST772 and ST672 elements code for a complete type I restriction modification system proteins hsdR, hsdS and hsdM. HsdR and hsdM are conserved among all ST772s and ST672 with respect to WIS and M013, while hsdS domain is different from that of WIS and M013 and has 80% similarity to bovine *S. aureus* LGA251 carrying SCC*mec* XI.

### Hypothetical Proteins

Several HPs from *S. aureus* 3957 and GR1 are not identified among the other six ST772 isolates but have 100% identity with M013 proteins. To our knowledge, ST 59 *S. aureus* isolates have not been detected in India but are present in Taiwan, China and Hong Kong. A common HA-MRSA present in India, China and Taiwan is ST239 which is the first bacterial hybrid to be found in nature [29]. We compared the nucleotide sequences (blastn) of SCC*mec* element of *S. aureus* 85/2082 (AB037671.1) with VH60, 3957 and GR1, and found 54%, 64%, and 67% query coverage respectively, with >97% identity. Blastp results indicate that 22/40, 21/39 and 10/28 proteins in GR1, 3957 and VH60 have more than 70% identity to 85/2082 SCC*mec* element proteins (Table S3). More specifically, 9 proteins between orfs 2-13 in GR1 and 3957 have more than 90% identity with 85/2082 and M013 proteins. It is likely that some of these proteins have originated from ST239 through horizontal transfer to generate SCC*mec* elements of ST772 and ST672. M013 SCC*mec* element has perhaps been generated through similar independent recombinations. HPs coded by orf 10 and orf 11 are homologous with SCC*mec* elements of ZH47 and *S. epidermidis* (CAL22884.1 and EJE32225.1). The last two HPs present in ST772 and ST672 SCC*mec* elements originate from *S. epidermidis* (ZP_04797658.1 and ZP_04797657.1) and are not present in WIS or M013.

SCC*mec* V element present in most Indian ST772 isolates is the smallest perhaps rendering the organism fittest to survive. The generation of Indian ST772 and ST672 type V SCC*mec* elements point to novel rearrangements due to recombination events (deletion/addition) involving other *S. aureus* including ST239 isolates, bovine SCC*mec* elements and elements from other Staphylococci.

## Materials and Methods

### Ethical Statement

The sequenced *S. aureus* and other ST772 isolates were obtained from clinicians from different hospitals in India. These hospitals have their own ethical boards which give them permission to collect these samples. M013 and WIS were obtained through the courtesy of Prof. Etienne, University of Lyon, France. The eight sequenced and 45 ST772 *S. aureus* isolates used to check the frequency of appearance of SCC*mec* elements similar to *S. aureus* 3957, were part of this collection.

The collection and molecular characterization of *S. aureus* isolates were carried out as described in earlier publications [16], [17]. Whole genome sequencing of two *S. aureus* isolates, one ST772 and one ST672 respectively, have been described earlier [22], [24]. Whole genome sequencing was performed using Illumina HiSeq-1000 sequencer. The raw reads obtained were assembled into contigs using VELVET [30], and gene predictions were made using GLIMMER 3.02 [31]. The relative arrangement of the SCC*mec* structural elements in different contigs was determined by BLASTP and TBLASTN [32]. The arrangement of contigs corresponding to SCC*mec* elements was determined by Mummer [33]. The sequences were validated and joined by performing overlapping PCRs (Figure S2) and Sanger sequencing (Figure S3). We used previously published chromosome- SCC*mec* junction sequences to identify the sequences of SCC*mec* elements present in ST772 and ST672 [10], [22], [24]. The SCC*mec* element annotations for all eight isolates have been deposited in the DNA database of Japan (DDBJ).

## Supporting Information

Figure S1 **ClustalW alignment of** ***ccrC1*** **(allele 5) of JCSC1435 with 3957(Orf28 and 29) and GR1 (Orf 29 and 30).**
(DOC)

Figure S2 **Verification of SCC** ***mec*** **contig sequences by overlapping PCRs.**
(DOC)

Figure S3 **Example of Sanger sequencing.**
(PDF)

Table S1 **Comparison of orfs from SCC** ***mec*** **elements of** ***S. aureus*** **VH60 and corresponding orthologs of other ST772, ST672 and reference strains.**
(XLS)

Table S2 **Length of IS431 transposases among sequenced ST772 and ST672 isolates.**
(DOCX)

Table S3 **Comparison of SCC** ***mec*** **element orfs of 85/2082 with corresponding orthologs of VH60, 3957 and GR1.**
(XLS)
