# Supplementary material for: Novel Rearrangements in the Staphylococcal Cassette Chromosome Mec Type V Elements of Indian ST772 and ST672 Methicillin Resistant Staphylococcus aureus Strains
Source: PLoS One. 2014 Apr 10;9(4):e94293. doi: 10.1371/journal.pone.0094293 (PMC3983117; doi:10.1371/journal.pone.0094293)
Supplement: Figure S1 — ClustalW alignment of ccrC1 (allele 5) of JCSC1435 with 3957(Orf28 and 29) and GR1 (Orf 29 and 30). (DOC) [file pone.0094293.s001.doc]

**Figure S2:** ClustalW alignment of ccrC1 (allele 5) of JCSC1435 with 3957(Orf28 and 29) and GR1 (Orf 29 and 30).

**a)**

3957 orf28 MKGKIALYSRVSTSEQSEHGYSEKEQEQLLIKEVMKNFPGYDYETYTDSGISGKNIEGRP 60

JCSC1435 MKGKIALYSRVSTSEQSEHGYSEKEQEQLLIKEVMKNFPGYDYETYTDSGISGKNIEGRP 60

************************************************************

3957 orf28 AMKRLLQDVKDNKIEIVLSWXGN------------------------------------- 83

JCSC1435 AMKRLLQDVKDNKIEIVLSWKLNRISRSMRDVFNIIHEFKEHGVGYKSISENIDTSNASG 120

******************** *

3957 orf28 ------------------------------------------------------------

JCSC1435 EVLVTMFGLIGSIERSTLASNVKMSMNAKARSGEAITGRVLGYKLSLNPLTQKNDLVIDE 180

3957 orf28 ------------------------------------------------------------

JCSC1435 NEAHIVREIFDLYLNHNKGLKAITTILNQKGYRTINQKPFSVFGVKYILNNPVYKGYVRF 240

3957 orf28 ------------------------------------------------------------

JCSC1435 NNHQNWAVQRRSGKSDKNDVILVKGKHEAIISEDVFDQVHEKLASKSFKPGRPIGGDFYL 300

3957 orf28 ------------------------------------------------------------

JCSC1435 RGLIKCPECGNNMVCRRTYYKTKKSKERTIKRYYICSLFNRSGSSACHSNAINAEVVERV 360

3957 orf28 ------------------------------------------------------------

JCSC1435 INVHLNRILSQPNVIKQIASSVIEELKQKHSKQTEIKYDIDSLEKQKAKVKTQQERLLEL 420

3957 orf28 ------------------------------------------------------------

JCSC1435 FLDDEMDSEMLKAKQSEMNQQLEVLDQQIKEAKQANQSQGDIPNFDKLKARLILMITRFS 480

3957 orf28 ------------------------------------------------------------

JCSC1435 LYLRKATPEAKNQLMKMLIDSIEITTDKQVKLVRYKIDESLIPQSLKKDWGSFFMPKFQF 540

3957 orf28 ------------------

JCSC1435 EIDGRNNYFIDQITTFTT 558

**b)**

3957 orf29 ------------------------------------------------------------

JCSC1435 MKGKIALYSRVSTSEQSEHGYSEKEQEQLLIKEVMKNFPGYDYETYTDSGISGKNIEGRP 60

3957 orf29 ---------------------MNRISRSMRDVFNIIHEFKEHGVGYKSISENIDTSNASG 39

JCSC1435 AMKRLLQDVKDNKIEIVLSWKLNRISRSMRDVFNIIHEFKEHGVGYKSISENIDTSNASG 120

:**************************************

3957 orf29 EVLVTMFGLIGSIERSTLASNVKMSMNAKARSGEAITGRVLGYKLSLNPLTQKNDLVIDE 99

JCSC1435 EVLVTMFGLIGSIERSTLASNVKMSMNAKARSGEAITGRVLGYKLSLNPLTQKNDLVIDE 180

************************************************************

3957 orf29 NEAHIVREIFDLYLNHNKGLKAITTILNQKGYRTINQKPFSVFGVKYILNNPVYKGYVRF 159

JCSC1435 NEAHIVREIFDLYLNHNKGLKAITTILNQKGYRTINQKPFSVFGVKYILNNPVYKGYVRF 240

************************************************************

3957 orf29 NNHQNWAVQRRSGKSDKNDVILVKGKHEAIISEDVFDQVHEKLASKSFKPGRPIGGDFYL 219

JCSC1435 NNHQNWAVQRRSGKSDKNDVILVKGKHEAIISEDVFDQVHEKLASKSFKPGRPIGGDFYL 300

************************************************************

3957 orf29 RGLIKCPECGNNMVCRRTYYKTKKSKERTIKRYYICSLFNRSGSSACHSNAINAEVVERV 279

JCSC1435 RGLIKCPECGNNMVCRRTYYKTKKSKERTIKRYYICSLFNRSGSSACHSNAINAEVVERV 360

************************************************************

3957 orf29 INVHLNRILSQPNVIKQIASSVIEELKQKHSKQTEIKYDIDSLEKQKAKVKTQQERLLEL 339

JCSC1435 INVHLNRILSQPNVIKQIASSVIEELKQKHSKQTEIKYDIDSLEKQKAKVKTQQERLLEL 420

************************************************************

3957 orf29 FLDDEMDSEMLKAKQSEMNQQLEVLDQQIKEAKQANQSQGDIPNFDKLKARLILMITRFS 399

JCSC1435 FLDDEMDSEMLKAKQSEMNQQLEVLDQQIKEAKQANQSQGDIPNFDKLKARLILMITRFS 480

************************************************************

3957 orf29 LYLRKATPEAKNQLMKMLIDSIEITTDKQVKLVRYKIDESLIPQSLKKDWGSFFMPKFNF 459

JCSC1435 LYLRKATPEAKNQLMKMLIDSIEITTDKQVKLVRYKIDESLIPQSLKKDWGSFFMPKFQF 540

**********************************************************:*

3957 orf29 VINVANENRIENLSLLPLF 478

JCSC1435 EIDGRNNYFIDQITTFTT- 558

*: *: *:::: :.

**c)**

GR1 orf29 MKGKIALYSRVSTSEQSEHGYSEKEQEQLLIKEVMKNFPGYDYETYTDSGISGKNIEGRP 60

JCSC1435 MKGKIALYSRVSTSEQSEHGYSEKEQEQLLIKEVMKNFPGYDYETYTDSGISGKNIEGRP 60

************************************************************

GR1 orf29 AMKRLLQDVKDNKIEIVLSWKLNRISRSMRDVFNIIHEFKEHGVGYKSISENIDTSNASG 120

JCSC1435 AMKRLLQDVKDNKIEIVLSWKLNRISRSMRDVFNIIHEFKEHGVGYKSISENIDTSNASG 120

************************************************************

GR1 orf29 EVLVTMFGLIGSIERSTLASNVKMSMNAKARSGEAITGRVLGYKLSLNPLTQKNDLVIDE 180

JCSC1435 EVLVTMFGLIGSIERSTLASNVKMSMNAKARSGEAITGRVLGYKLSLNPLTQKNDLVIDE 180

************************************************************

GR1 orf29 NEAHIVREIFDLYLNHNKGLKAITTILNQKGYRTINQKPFSVFGVKYILNNPVYKGYVRF 240

JCSC1435 NEAHIVREIFDLYLNHNKGLKAITTILNQKGYRTINQKPFSVFGVKYILNNPVYKGYVRF 240

************************************************************

GR1 orf29 NNHQNWAVQRRSGKSDKNDVILVKGKHEAIISEDVFDQVHEKLASKSFKPGRPIGGDFYL 300

JCSC1435 NNHQNWAVQRRSGKSDKNDVILVKGKHEAIISEDVFDQVHEKLASKSFKPGRPIGGDFYL 300

************************************************************

GR1 orf29 RGLIKCPECGK------------------------------------------------- 311

JCSC1435 RGLIKCPECGNNMVCRRTYYKTKKSKERTIKRYYICSLFNRSGSSACHSNAINAEVVERV 360

**********:

GR1 orf29 ------------------------------------------------------------

JCSC1435 INVHLNRILSQPNVIKQIASSVIEELKQKHSKQTEIKYDIDSLEKQKAKVKTQQERLLEL 420

GR1 orf29 ------------------------------------------------------------

JCSC1435 FLDDEMDSEMLKAKQSEMNQQLEVLDQQIKEAKQANQSQGDIPNFDKLKARLILMITRFS 480

GR1 orf29 ------------------------------------------------------------

JCSC1435 LYLRKATPEAKNQLMKMLIDSIEITTDKQVKLVRYKIDESLIPQSLKKDWGSFFMPKFQF 540

GR1 orf29 ------------------

JCSC1435 EIDGRNNYFIDQITTFTT 558

**d)**

GR1 orf30 ------------------------------------------------------------

JCSC1435 MKGKIALYSRVSTSEQSEHGYSEKEQEQLLIKEVMKNFPGYDYETYTDSGISGKNIEGRP 60

GR1 orf30 ------------------------------------------------------------

JCSC1435 AMKRLLQDVKDNKIEIVLSWKLNRISRSMRDVFNIIHEFKEHGVGYKSISENIDTSNASG 120

GR1 orf30 ------------------------------------------------------------

JCSC1435 EVLVTMFGLIGSIERSTLASNVKMSMNAKARSGEAITGRVLGYKLSLNPLTQKNDLVIDE 180

GR1 orf30 ------------------------------------------------------------

JCSC1435 NEAHIVREIFDLYLNHNKGLKAITTILNQKGYRTINQKPFSVFGVKYILNNPVYKGYVRF 240

GR1 orf30 ------------------------------------------------------------

JCSC1435 NNHQNWAVQRRSGKSDKNDVILVKGKHEAIISEDVFDQVHEKLASKSFKPGRPIGGDFYL 300

GR1 orf30 ------------MVCRRTYYKTKKSKERTIKRYYICSLFNRSGSSACHSNAINAEVVERV 48

JCSC1435 RGLIKCPECGNNMVCRRTYYKTKKSKERTIKRYYICSLFNRSGSSACHSNAINAEVVERV 360

************************************************

GR1 orf30 INVHLNRILSQPNVIKQIASSVIEELKQKHSKQTEIKYDIDSLEKQKAKVKTQQERLLEL 108

JCSC1435 INVHLNRILSQPNVIKQIASSVIEELKQKHSKQTEIKYDIDSLEKQKAKVKTQQERLLEL 420

************************************************************

GR1 orf30 FLDDEMDSEMLKAKQSEMNQQLEVLDQQIKEAKQANQSQGDIPNFDKLKARLILMITRFS 168

JCSC1435 FLDDEMDSEMLKAKQSEMNQQLEVLDQQIKEAKQANQSQGDIPNFDKLKARLILMITRFS 480

************************************************************

GR1 orf30 LYLRKATPEAKNQLMKMLIDSIEITTDKQVKLVRYKIDESLIPQSLKKDWGSFFMPKFNF 228

JCSC1435 LYLRKATPEAKNQLMKMLIDSIEITTDKQVKLVRYKIDESLIPQSLKKDWGSFFMPKFQF 540

**********************************************************:*

GR1 orf30 VINVANENRIENLSLLPLF 247

JCSC1435 EIDGRNNYFIDQITTFTT- 558

*: *: *:::: :.
