# Supplementary material for: Novel Rearrangements in the Staphylococcal Cassette Chromosome Mec Type V Elements of Indian ST772 and ST672 Methicillin Resistant Staphylococcus aureus Strains
Source: PLoS One. 2014 Apr 10;9(4):e94293. doi: 10.1371/journal.pone.0094293 (PMC3983117; doi:10.1371/journal.pone.0094293)
Supplement: Figure S2 — Verification of SCC mec contig sequences by overlapping PCRs. (DOC) [file pone.0094293.s002.doc]

**S5:**

**Verification of SCC*mec* contig sequences by over lapping PCRs and Sanger sequencing:**

The SCC*mec* elements of all the sequenced isolates were mostly present in 2 or 3 contigs. Multiple or repeated assembly with different assemblers (Velvet, Soapdenovo) have provided the same result. Gene prediction using different softwares (Glimmer, GeneMark) gave the same number of orfs.

We have done several over lapping PCRs to connect the contigs and verify the sequences obtained from whole genome sequences by Sanger sequencing.

Examples of PCR verifications: 1) SCC*mec* region in 118 was split into two contigs after velvet assembly of the raw sequence reads. PCR was done to connect the two contigs by designing primers between orf 2 and orf 3 and product was sequenced (Fig 1A and SM5).

**Figure 1**

**A B C D**


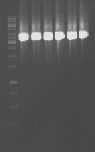

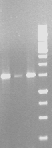


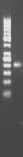


196bp

3.1kb

1.2kbp

1.5kb


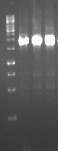


2) The SCC*mec* elements of five ST772 isolates (VH60, 120, 3989, 333 and LVP2) were split into 2 or 3 contigs after velvet assembly and the overall arrangement of SCC*mec* genes were similar to that of 118. Their arrangements were reconfirmed by 3 sets o PCRs -PCR1 to link *orfX* (orf 1) to PBP2a (orf 4); PCR2 - HMG-CoA (orf 8) to hypothetical protein (orf 10); PCR3- 2 Hypothetical proteins orf 27 to orf 28. The product sizes were as determined for 118 sequences, and were 3.2 kb, 1.6 kb and 1.4 kb respectively for three PCRs (Picture not shown).

3) SCC*mec* in GR1 (ST672) and 3957 (ST772) were split into 4 contigs after the velvet assembly of the raw sequence reads. Three PCRs were done to connect and validate the arrangement of nodes in the SCC*mec* of GR1 and 3957 as shown in figs B: orf 13(HP) to orf 15(PBP2a); C: orf 19(HMG-CoA) to orf 21(HP); D: orf 39(HP) to orf 40(HP).

4) Split ccrCs in 3957 and GR1 were reconfirmed by Sanger sequencing (chromotogram not shown. ClustalW alignments of the sequences are in Fig S2).
