# Supplementary material for: Novel Rearrangements in the Staphylococcal Cassette Chromosome Mec Type V Elements of Indian ST772 and ST672 Methicillin Resistant Staphylococcus aureus Strains
Source: PLoS One. 2014 Apr 10;9(4):e94293. doi: 10.1371/journal.pone.0094293 (PMC3983117; doi:10.1371/journal.pone.0094293)

File: 118_SCCmec_118_SCCmecF.ab1 *Run Ended: 2012/11/14 18:42:16* *Signal G:7647 A:13886 C:15746 T:19391*

Sample: 118_SCCmec_118_SCCmecF *Lane: 12* *Base spacing: 15.061864* *580 bases in 6717 scans*

10 20 30 40 50 60 70 80 90 100 110 120
TTTT TTT TT C GGT CCGAA GTAAT GGG GTT CTT GTT GCAAA GTAAAAAAATATA GCTAACCACTAATTT AT CAT GT CAG TGTTTCGCTTAACT TGCTAGCATGAT GCTAATTTTCG TGGCATGGC G

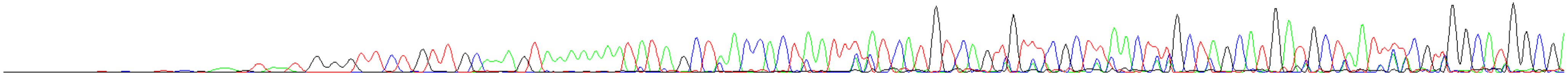

130 140 150 160 170 180 190 200 210 220 230 240 250
AAAATTCCGTAGATCTGAAGAGACCTGCGGTTCTTTTTATATAGAGCGTAAATACATTTAATACCTTTTAAAGTATTCTTTGCTGTATTGATACTTTGATACCTTGTCTTTCTTACTTTAATATGACGGTG

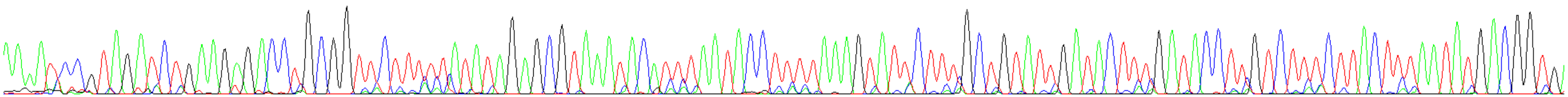

260 270 280 290 300 310 320 330 340 350 360 370 380
ATCTTGCTCAATGAGGTTATTCAGATATTTCGATGTACAATGACAGTCAGGTTTAAGTTTAAAACCTTTAATTACTTTAGCCATTGCTACCTTCGTTGAAGGTGCCTGATCTGTAATTACCTTTTGAGGTT

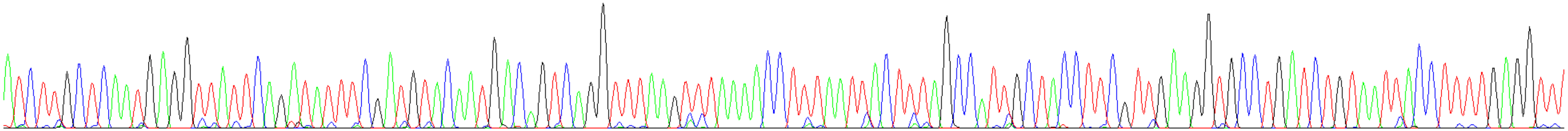

390 400 410 420 430 440 450 460 470 480 490 500 510 520
TACCAAATTGTTTAATGAGACGTTTGATAAACGCATATGCTGAATGATTATCTCGTTGCTTACGCAACCAAATATCTAATGTATATCCCTCTGCATCAATGGCACGATATAAATAGCTCCATTTTCCTTTTATTT

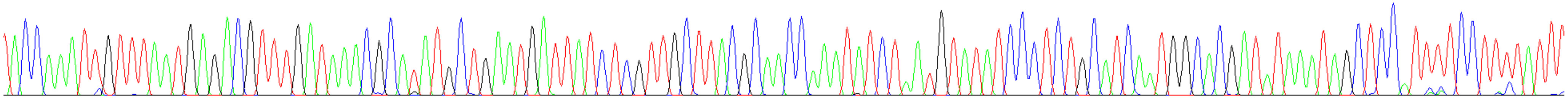

530 540 550 560 570 580
TGATGT CGTCTCA CA TAC CCTG GGAATGTGTTTTTTTTAATTTAATTATGGTTTTTTTT

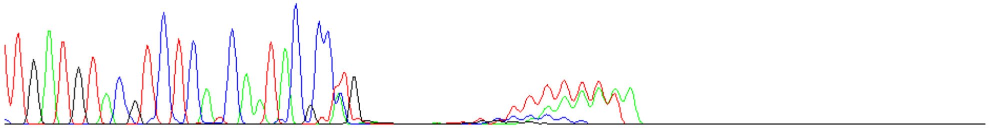

File: 118_SCCmec_118_SCCmecR.ab1 Run Ended: 2012/11/14 22:38:15 Signal G:5728 A:14026 C:14010 T:16252

Sample: 118_SCCmec_118_SCCmecR Lane: 12 Base spacing: 14.818626 549 bases in 6656 scans Page 1 of 1

MACROGEN
Advancing through Genomics

10 20 30 40 50 60 70 80 90 100 110 120

TT TTT TT A T AAT CC GTT GGC CAT TT G ATT GCA GAAG G GAT AT ACATT T A GAT AT T TCG GT TGC GTAAGCAACGAGATAATCATTCAGCATATGCGTTTATCAAACGTCTCATTAAACAATTTG

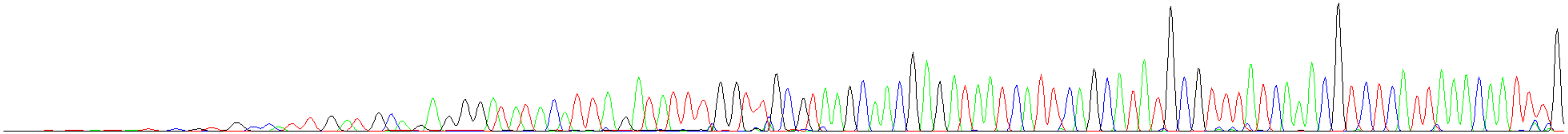

130 140 150 160 170 180 190 200 210 220 230 240

GTAAACCTCAAAAGGTAATTACAGATCAGGCACCTTCAACGAAGGTAGCAATGGCTAAAGTAATTAAAGGTTTTAAACTTAAACCTGACTGTCATTGTACATCGAAATATCTGAATAACCTCAT

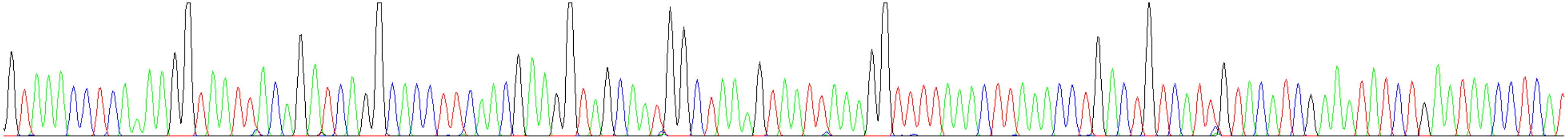

250 260 270 280 290 300 310 320 330 340 350 360 370

TGAGCAAGATCACCGTCATATTAAAGTAAGAAAGACAAGGTATCAAAGTATCAATACAGCAAAGAATACTTTAAAAGGTATTAAATGTATTTACGCTCTATATAAAAAGAACCGCAGGTCTCTTC

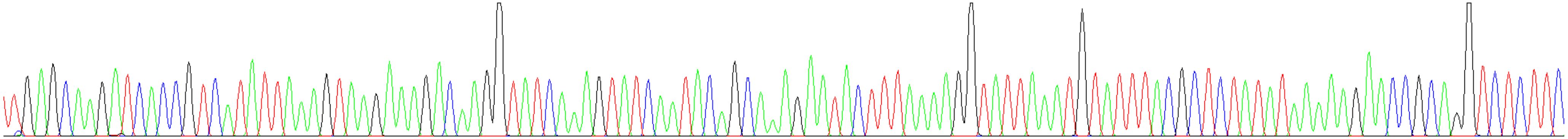

380 390 400 410 420 430 440 450 460 470 480 490

AGATCTACGGATTTTCGCCATGCCACGAAATTAGCATCATGCTAGCAAGTTAAGCGAACACTGACATGATAAATTAGTGGTTAGCTATATTTTTTTACTTTGCAACAGAACCCATTACTTCGA

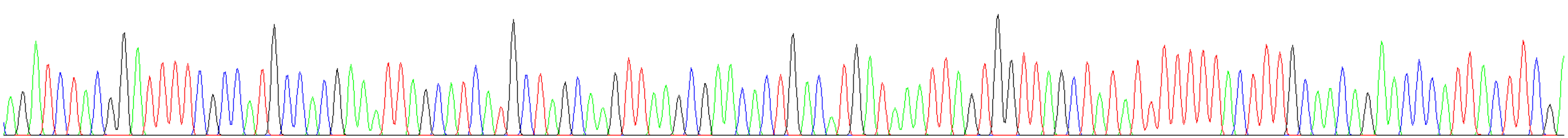

500 510 520 530 540

CGAATAATCTTACCTCCTCTTTTAGCTACTTCTTTTCCTATTTGAAAAGTTTTG

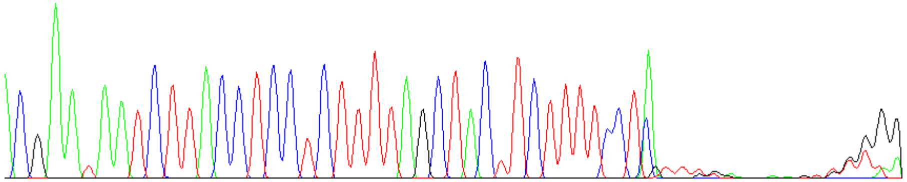

Supplement: Figure S3 — Example of Sanger sequencing. (PDF) [file pone.0094293.s003.pdf]
