# Supplementary material for: Novel Rearrangements in the Staphylococcal Cassette Chromosome Mec Type V Elements of Indian ST772 and ST672 Methicillin Resistant Staphylococcus aureus Strains
Source: PLoS One. 2014 Apr 10;9(4):e94293. doi: 10.1371/journal.pone.0094293 (PMC3983117; doi:10.1371/journal.pone.0094293)
Supplement: Table S2 — Length of IS431 transposases among sequenced ST772 and ST672 isolates. (DOCX) [file pone.0094293.s005.docx]

Table S3: **Length of IS431 transposases among sequenced**

**ST772 and ST672 *S. aureus* isolates**

| **Strain/Isolate** | **Orf/ IS431 transposase length** |
| --- | --- |
| VH60 | Orf 3/ 92aa  Orf 9/ 48aa |
| 118 | Orf 3/ 92aa  Orf 8/ 48aa |
| 120 | Orf 4/92aa  Orf 9/157aa |
| 333 | Orf 3/ 133aa  Orf 8/ 48aa |
| 3989 | Orf 3/ 92aa  Orf 8/ 48aa |
| LVP2 | Orf 3/ 155aa  Orf 8/ 48aa |
| 3957 | Orf 14/ 223aa  Orf 20/ 440aa (transposase for 1181) |
| GR1 | Orf 15/ 128aa  Orf 21/ 128aa |
